# Red blood cell blood group A antigen level affects the ability of heparin and PfEMP1 antibodies to disrupt *Plasmodium falciparum* rosettes

**DOI:** 10.1186/s12936-021-03975-w

**Published:** 2021-11-18

**Authors:** Pontus Hedberg, Madle Sirel, Kirsten Moll, Mpungu Steven Kiwuwa, Petter Höglund, Ulf Ribacke, Mats Wahlgren

**Affiliations:** 1 Department of Microbiology, Tumor and Cell Biology (MTC), Karolinska Institutet, 171 65 Stockholm, Sweden; 2 Department of Infectious Diseases, Karolinska University Hospital, 171 76 Stockholm, Sweden; 3 Department of Medicine, Huddinge, Karolinska University Hospital, 141 86, Stockholm, Sweden; 4 Department of Child Health and Development Centre, School of Medicine, Makerere University College of Health Sciences, Kampala, Uganda

**Keywords:** Malaria, *Plasmodium falciparum*, Rosetting, Histo-blood group ABO system

## Abstract

**Background:**

The histo-blood group ABO system has been associated with adverse outcomes in COVID-19, thromboembolic diseases and *Plasmodium falciparum* malaria. An integral part of the severe malaria pathogenesis is rosetting, the adherence of parasite infected red blood cells (RBCs) to uninfected RBCs. Rosetting is influenced by the host’s ABO blood group (Bg) and rosettes formed in BgA have previously been shown to be more resilient to disruption by heparin and shield the parasite derived surface antigens from antibodies. However, data on rosetting in weak BgA subgroups is scarce and based on investigations of relatively few donors.

**Methods:**

An improved high-throughput flow cytometric assay was employed to investigate rosetting characteristics in an extensive panel of RBC donor samples of all four major ABO Bgs, as well as low BgA expressing samples.

**Results:**

All non-O Bgs shield the parasite surface antigens from strain-specific antibodies towards *P. falciparum* erythrocyte membrane protein 1 (PfEMP1). A positive correlation between A-antigen levels on RBCs and rosette tightness was observed, protecting the rosettes from heparin- and antibody-mediated disruption.

**Conclusions:**

These results provide new insights into how the ABO Bg system affects the disease outcome and cautions against interpreting the results from the heterogeneous BgA phenotype as a single group in epidemiological and experimental studies.

**Graphical Abstract:**

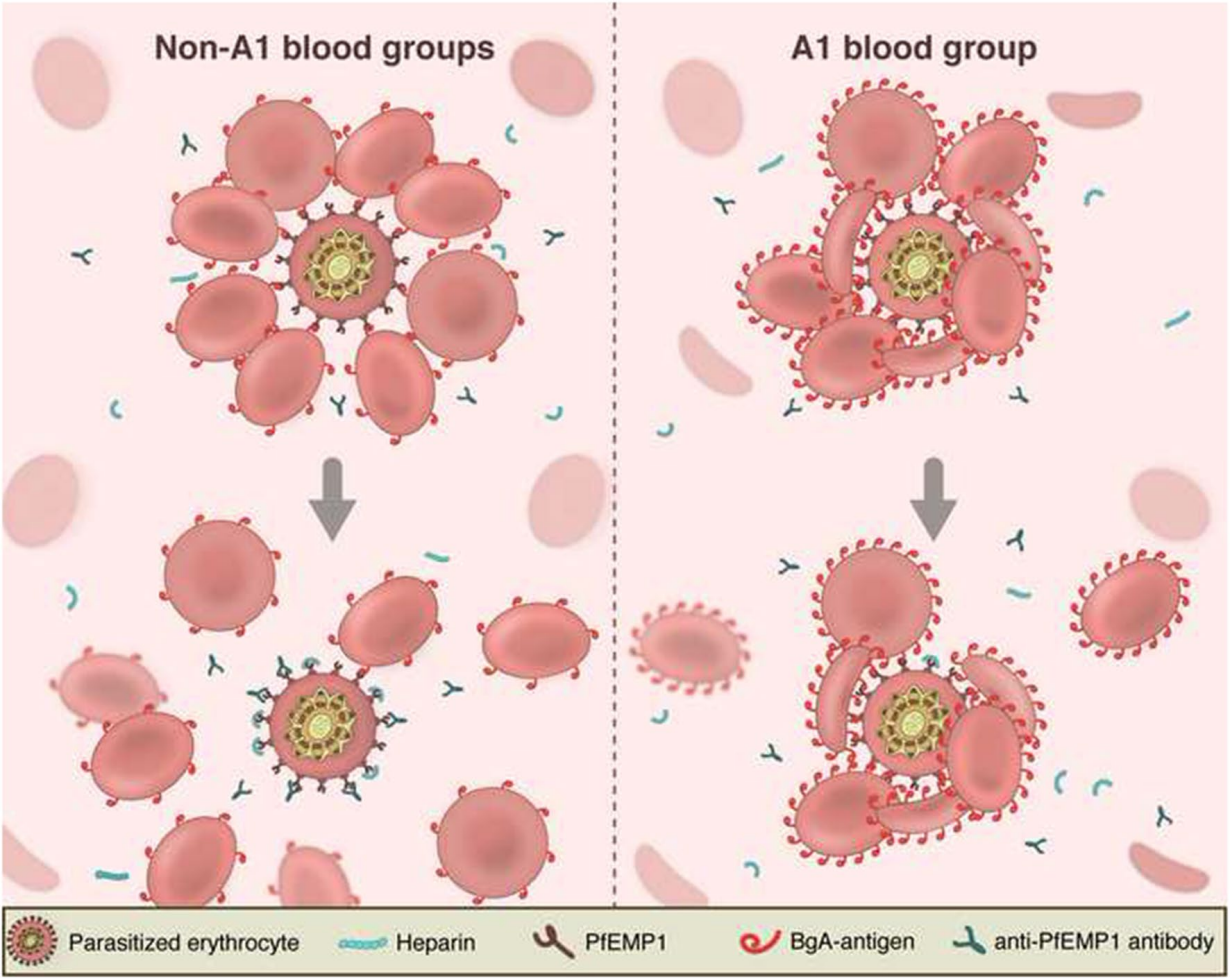

**Supplementary Information:**

The online version contains supplementary material available at 10.1186/s12936-021-03975-w.

## Background

Severe *Plasmodium falciparum* malaria has exerted an enormous mortality toll over the course of human evolution and as of today, the disease still causes approximately 400,000 annual deaths [1].

Severe malaria (SM) is a heterogeneous disease state with a multifaceted and poorly understood pathogenesis [2]. Besides a wide range of parasite factors, several human host genetic factors have been shown to influence the susceptibility to SM, including the sickle-cell trait, α^+^-thalassaemia and the histo-blood group (Bg) ABO system [3–6]. Consequently, many of these have been evolutionary selected for in human populations in malaria endemic regions, which also highlights the influence this disease has on the trade-off between risk and benefit for human adaptation [7].

Epidemiological and genome-wide association studies have provided robust evidence of a protective effect of BgO as compared to non-O Bgs with regards to development of SM [5, 6, 8, 9]. SM has also been strongly linked to the ability of *P. falciparum* iRBCs to bind to uninfected RBCs and the vascular endothelium, cytoadhesive events known as rosetting and cytoadherence, respectively [10, 11]. These phenomena collectively contribute to blockade of oxygen transport in the microvasculature and endothelial activation with an augmented inflammatory response, all hallmark features of the disease pathobiology [10]. Rosetting is mediated by PfEMP1, RIFIN and STEVOR, parasite ligands that interact with a plethora of RBC receptors such as BgA, complement receptor 1 (CR1) and glycophorin C (GYPC) [12–15]. BgA rosetting has been shown to be more prominent compared to BgO, with augmented rosetting rates (RR) and rosette sizes (RS) [6, 13, 16]. Further, BgA-mediated rosetting has been suggested to confer protection from the immune system of the host, by preventing exposure of important immunogenic parasite-derived epitopes at the RBC surface [17].

BgA has more extensive, genetically driven, variation in erythrocytic antigen expression as compared to BgB, with A1 RBCs possessing approximately five times more A antigen than A2 RBCs [18]. Similarly, BgAB can be divided into A1B and A2B depending on the A-transferase allele [19]. Although there is a general consensus of A antigens promoting cytoadhesive events, the data on rosetting in weak versus strong BgA subgroups is scarce and based on investigations of relatively few donors [13, 16, 20]. In addition, besides host cell resident ABO antigens, differences in serum component levels, such as soluble ABO antigens, von Willebrand Factor, ICAM-1 and E-selectin exist and their relative contribution in the context of Bgs is not well understood [21–23].

A better understanding of how host factors are involved in the cytoadhesive events implicated in SM pathogenesis is warranted. The current paper explores the effect of all four ABO Bgs on *P. falciparum* rosetting, with a particular emphasis on low A antigen expression levels.

## Methods

### Parasite cultures

The parasite lines used in this study were the two laboratory parasite clones FCR3S1.2 and PaloAltoVarO (PAvarO) and four culture-adapted clinical isolates, UCM83, UKM74, UKM104 and UKS111 collected in Kampala, Uganda [24, 25]. All parasite lines were cultivated according to standard procedures [26, 27]. Briefly, all cultures were grown in malaria culture medium (MCM) (RPMI 1640 (Gibco) supplemented with 2 mM l-glutamine (Hyclone), 2.5 µg/mL gentamicin (Gibco), bicarbonate at final concentration of 32 mM (Sigma) and 10% A + serum) at 4% haematocrit under constant microaerophilic condition (5% O_2_, 5% CO_2_, 90% N_2_) and in suspension on an orbital shaker. Prior to assaying, parasites were propagated in O+ erythrocytes with 10% AB+ serum for the laboratory clones and 15% AB+ serum for the patient isolates.

### Sample collection

All RBC and serum samples used in the study were obtained from healthy Swedish blood donors. For RBC samples, intravenous blood was collected in Vacutainer® citrate tubes (BD) and serum samples were collected into Vacutainer® SST™II Advance gel tubes (BD). Aliquots of sera were stored at − 20 °C and heat inactivated at 56 °C for 30 min prior to use.

### ABO antigen semi-quantification

Subdivision of A1-negative and A1-positive BgA RBCs was performed using anti-A1 lectin (Ortho Clinical Diagnostics, catalogue number 711830) at the site of collection. This serological classification was not available for the BgAB samples, since this was not part of routine clinical practice. The semi-quantification of A and B antigens by flow cytometry was performed as previously described, with 10,000 RBCs acquired per sample [28]. In brief, RBCs were blocked in 2% bovine serum albumin (fraction V, HyClone) in PBS (BSA-PBS) for 5 min at room temperature. Thereafter primary antibodies (anti-BgA (sc-52367) and anti-BgB (sc-52371, Santa Cruz Biotechnology) were added at 10 µg/mL for 10 min at room temperature. After two washes with PBS, FITC conjugated secondary anti-mouse IgM (μ-chain specific) (Sigma, F9259) was used at 1:10 dilution in 2% BSA-PBS for 10 min at room temperature. Lastly, RBCs were washed with PBS and resuspended in PBS for acquisition.

### Rosetting assays

For rosetting assays involving RBCs of different Bgs, late stage parasite cultures were diluted 1:25 in donor RBCs in triplicates in 96-well plates for two cell cycles, with a desired final parasitaemia of 5–7.5%. For serum rosetting assays, parasites were grown in BgO RBCs in 10% of each serum for one cell cycle in triplicates. All samples were kept at 1% haematocrit. The rosetting assays were either investigating the innate rosetting characteristics or the rosette-disrupting propensity in different ABO Bgs. To evaluate rosette disruption by heparin and PfEMP1-DBL1α-antibodies (anti-var60 and anti-varO) [29], parasite cultures were incubated either with heparin (from Porcine intestine, Sigma, H3393) or antibodies at concentrations indicated for specific experiments at 37 °C for 45 min in MCM and measured by flow cytometry. For determination of rosetting rate (RR) and rosette size (RS) by microscopy, parasite cultures were stained with acridine orange [26]. The RR of 150–200 late stage iRBCs was counted for each sample and the number of bound RBCs in 100 rosettes were counted to determine RS.

### Flow cytometric analysis of rosetting rate and rosette size

The flow cytometric assays were performed using FACSVerse with universal loader (BD Bioscience). The cuvette cross section of this machine is 430 μm × 180 μm and data was acquired at medium flow rate (60 µl/min) with sheath core stream fluid velocity of 5.4 m/s. Samples were kept in suspension between the reads by 5 s shaking at 1400 rpm every 10 min. To discriminate between rosetting and non-rosetting late stage iRBCs, cells were co-stained with 10 μg/mL Hoechst 33,342 (Invitrogen, H3570) and 5 μg/mL Dihydroethidium (DHE, Invitrogen, D1168) in RPMI 1640 (HyClone) for 45 min at 37 °C. As described previously [30, 31]. The percentage of multiplets (events represented by 2 cells or more) after gating on this late-stage parasite population has previously been shown to correlate with RR determined by microscopy [31]. This gating strategy was adopted, with further optimizations, in order to enable absolute quantification of RR, as well as relative comparison of RS. Briefly, the initial FSC-SSC gating was excluded in order to include larger multiplets. Key to successful absolute quantification of RR and relative measures of RS was to allow larger multiplets to be identified, which simply accommodates inclusion of larger rosetting events. For this, voltages were adjusted so the cell population would be located closer to the lower left quadrant in the SSC-A–FSC-A plot. This resulted in tube target values of approximately 0.09 for FSC and 0.75 for SSC, which is on average 30% lower than for the previously described assay.

The gating was performed in the following order: Double-positive events for Hoechst and DHE (trophozoites and schizonts), FSC-A vs FSC-H for multiplets to determine RR based on the uninfected RBCs from the same sample and from there the mean SSC-A of multiplets for RS (Fig. 2A–C). Data from 1000–5000 late stage iRBCs were collected for the assays, depending on numbers and parasitaemias of samples.

### Antibody recognition of PfEMP1

Antibody recognition of PfEMP1 on iRBCs cultured in the four major ABO Bgs were performed using strain-specific PfEMP1-DBL1α-antibodies according to previously described methods [17]. Briefly, late-stage FCR3S1.2 and PAvarO cultures were incubated in 2% BSA-PBS for 45 min, followed by 45 min of incubation with strain-specific antibodies at 10 μg/mL or non-immune goat IgG 10 μg/mL. Thereafter, samples were washed with 2% BSA-PBS and incubated for 45 min with Alexa-488 coupled rabbit anti-goat IgG antibodies (1:100) (Invitrogen, A11078) and Hoechst 33342 (10 μg/mL) (Invitrogen, H3570) in 2% BSA-PBS. The samples were then washed three more times in 2% BSA-PBS and resuspended in 2% BSA-PBS with subsequent flow cytometric cell acquisition. All steps were done at 37 °C. Data was acquired with a flow cytometer as described above. The production and purification of anti-var60 (from strain FCR3S1.2), anti-varO (PAvarO) and non-immune polyclonal ChromPure goat IgG (Jackson Immuno Research, 005-000-003) used in this work was previously described in [29].

### Extraction of ABO allele frequencies from the Erythrogene database

To retrieve detailed ABO blood group allele frequencies, the Erythrogene database (https://www.erythrogene.com/) was used, containing next-generation sequencing data for all 36 blood group alleles in the 1000 Genomes Project [32]. 235 alleles were retrieved upon searching for ABO alleles, for which the ABO phenotype was classified into O, A1, A2, Aweak, Ax/Aweak, B, Bweak, B(A), B3 and unknown (previously uncharacterized ABO alleles). The results were analysed and displayed per continent (Africa, America, East Asia, Europe, South Asia).

### Data analysis and statistics

The cell acquisition was done using FACSVerse (BD Bioscience) flow cytometer and data was analysed using FlowJo (v10). All statistical analyses were performed in R version 4.0.3. Unpaired two-tailed t test was used for all pairwise comparisons of RR, RS and the relative mean fluorescence intensity (MFI) between different ABO Bgs, with p-values adjusted for multiple comparisons using the Holm method. Spearman rank’s correlation test was used for correlation testing.

### Ethics statement

All RBC and plasma samples used were collected from the Karolinska University Hospital Blood Bank and approved by the Regional Ethical Review Board in Stockholm, Sweden (Dnr 2009/668-31/3). The collection of clinical isolates in Uganda was approved by Karolinska Institute’s Regional Ethical Review Board (permission 03/095) and the Uganda National Council for Science and Technology (permission MV717). Written informed consent was obtained from the parents or guardians of the patients.

## Results

### ABO allele frequencies in the 1000 Genomes project

**Fig. 1:**
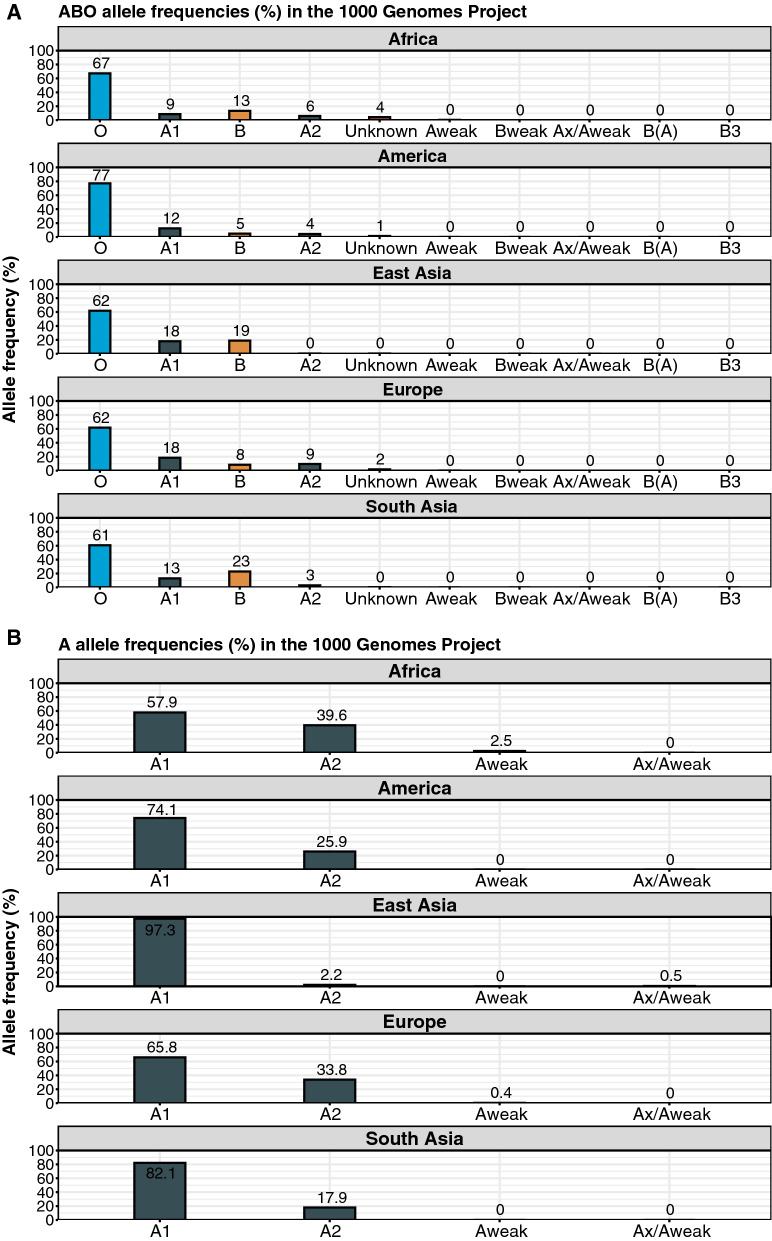
ABO allele frequencies in the 1000 Genomes project. **A** The ten most common ABO-alleles in the five population cohorts from 1000 Genomes project data and their corresponding allele frequencies. **B** Distribution of BgA alleles within the total BgA allele pool per population cohort from 1000 Genomes project data

The Erythrogene database containing data on Bg genes and allele frequencies from the 1000 Genomes project was mined to elucidate the relative frequencies of ABO Bgs among human populations [32]. As expected, BgO was the most dominant Bg globally, with an allele frequency ranging from 61 to 77% (Fig. 1A). The African cohort, which also experiences the highest burden of *P. falciparum* malaria, had the lowest BgA allele frequency, with 42% of the BgA allele frequency being represented by low-expressing BgA alleles (predominantly A2) (Fig. 1B). Corresponding figures for the American, East Asian, European and South Asian population cohorts were 26%, 2%, 34% and 18%, respectively (Fig. 1A). Although these data are based on a limited sample size and sensitive to confounders, this highlights the possibility of an evolutionary pressure for low A antigen BgA in regions highly endemic for *P. falciparum* malaria.

### An improved flow cytometric method for measuring *P. falciparum* rosetting

**Fig. 2:**
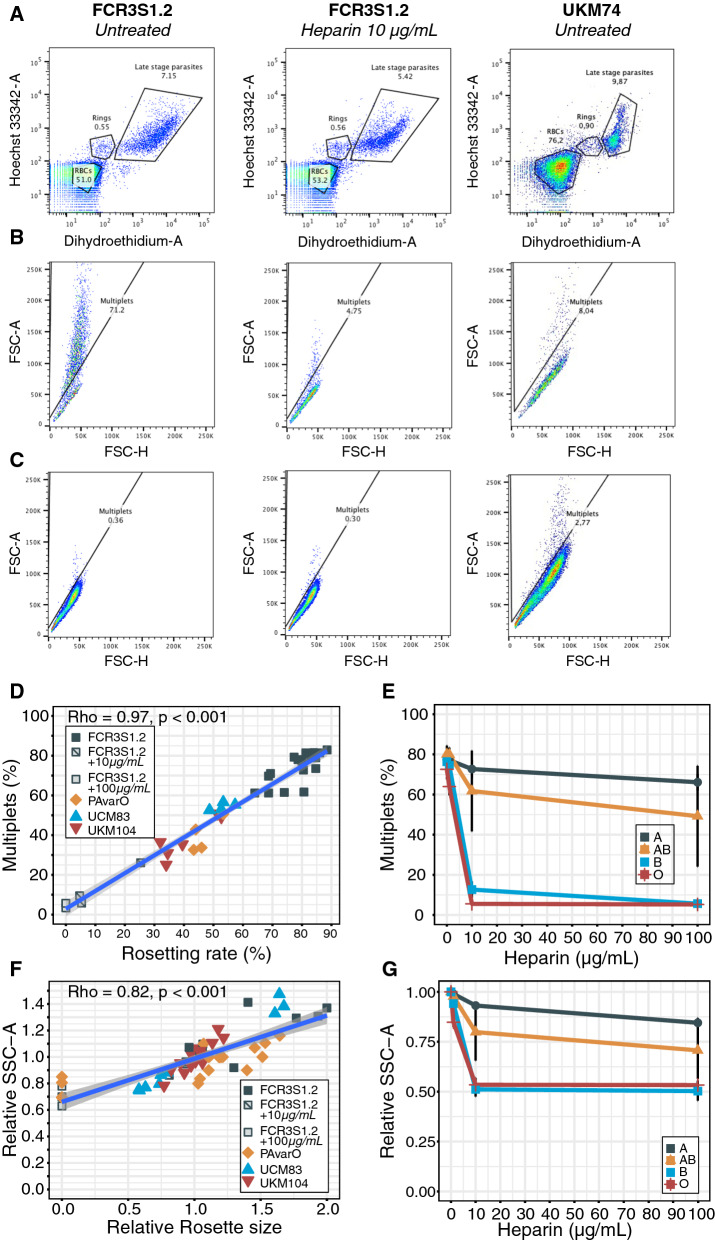
An improved flow cytometric method for measuring *P. falciparum* absolute rosetting rate and relative rosette size. **A** Parasite cultures were co-stained with Hoechst and Dihydroethidium (DHE) to differentiate uninfected RBCs from Hoechst positive ring and double positive late stage parasite (trophozoite and schizonts) infected RBCs in untreated (left panels), 10 µg/mL heparin-treated (middle panels) and low rosetting UKM74 (right panels) samples. **B** The percentage of multiplets of late parasite stage was determined by gating for events that did not have a proportional forward scatter area (FSC-A) to forward scatter height (FSC-H) ratio based on **C** the uninfected RBCs from the same samples. **D** The percentage of multiples from FCR3S1.2 (± 10 or 100 µg/mL heparin), PAvarO, UCM 83, UKM104 parasite cultures with variable RR was correlated to RR determined by microscopic enumeration of 150–200 iRBCs using Spearman’s rank correlation coefficient. **E** FCR3S1.2 parasites were grown in the blood of seven donors from each blood group and treated with heparin prior to flow cytometry. Percentage of multiplets upon addition of heparin in BgO (crosses), BgA (circles), BgB (squares) and BgAB (triangles) (mean ± SD). **F** Multiplets relative mean side scatter area (SSC-A) was correlated to the rosette sizes determined under the microscope by counting the mean number of RBC bound to iRBC (100 iRBCs/sample). Correlation was measured using Spearman’s rank correlation coefficient. All samples were normalized to the mean SSC-A in BgO, which was set as 1. **G** FCR3S1.2 parasites were grown in the blood of seven donors from each blood group and treated with heparin prior to flow cytometry. Relative multiplet size (SSC-A) upon addition of heparin in BgO (crosses), BgA (circles), BgB (squares) and BgAB (triangles) (mean ± SD). The mean SSC-A without heparin was set as 1 for each blood group

In order to rigorously determine the relevance of low A antigen in relation to *P. falciparum* rosetting, an improvement of existing methodologies was needed. Despite good correlations between RR and percentage of multiplets detected by microscopy and flow cytometry respectively, the previously developed method failed to determine absolute RR and thereby precluded identification of subtler, yet important, differences in RR [31]. Here, the voltages for FSC and SSC were lowered and the first gating on FSC-SSC was eliminated, thereby including all rosetting events (Fig. 2A–C). In addition, the SSC-A was utilized as indicative measurement for the relative RS. With this approach there was a strong and near perfect positive correlation between the RR and the percentage of multiplets (ρ = 0.97, *P* < 0.001) over a wide range (0 to 89% and 3 to 83%, respectively) (Fig. 2D). In order to further validate the sensitivity of the method, the parasite line FCR3S1.2, with known sensitivity to heparin-induced rosette dispersion in BgO but relative resistance in BgA, was selected. This parasite line was propagated in RBCs of 28 different donors (seven from each blood group) and exposed to 0, 1, 10 and 100 μg/mL of heparin before analysed by flow cytometry. In BgO, the mean rosetting was 73% (95% CI 70–75) without heparin and 6% (95% CI 5–6) with 10 μg/mL, whereas the corresponding mean RR for BgA was 77% (95% CI 75–80) and 73% (95% CI 70–75), respectively (Fig. 2E). These findings are in line with previously reported FCR3S1.2 rosetting phenotypes in BgO and BgA [17] and demonstrate the assay’s sensitivity to detecting changes in RR.

Regarding RS, the mean SSC-A of late stage multiplets was compared to the RS scored by microscopy and also here a positive correlation was observed (ρ = 0.82, *P* < 0.001), albeit with an apparent lower dynamic range of the flow cytometry approach (Fig. 2F). Thereafter, the sensitivity of the assay to detect heparin mediated reduction in RS was investigated. In BgO, the mean relative SSC-A was reduced by 47% (95% CI 44–50) in 10 μg/mL as compared to 0 μg/mL (Fig. 2G). For BgA, BgB and BgAB, the corresponding mean relative reduction was 7% (95% CI 6–8%), 49% (95% CI 46–52) and 20% (95% CI 7–33%), respectively (Fig. 2G).

### Blood group A and B influences parasite rosetting in a strain-dependent manner

**Fig. 3:**
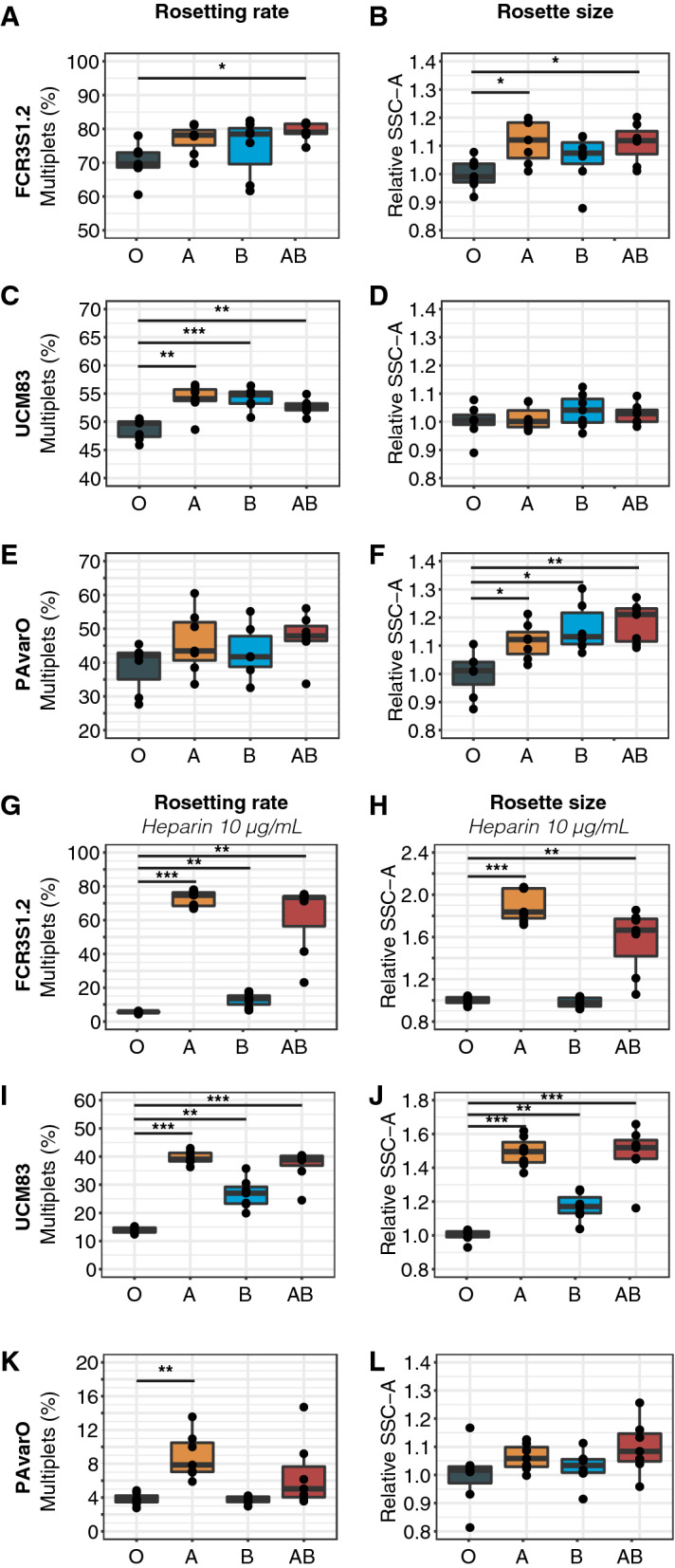
Blood group A and B influences parasite rosetting in a strain-dependent manner. Two laboratory strains (FCR3S1.2, PAvarO) and one Ugandan patient isolates (UCM83) were grown in RBCs from seven donors per blood group and rosetting characteristics determined by flow cytometry. Rosetting rate (left panel, percentage of multiplets) and rosette size (right panel, relative SSC-A) was measured without (**A**-**F**) and with (**G-L**) the addition of 10 µg/mL of heparin. Pairwise comparison of BgO and all non-O Bgs performed using unpaired t-test with Holm adjustment of P-values which are indicated with asterisks (* P ≤ 0.05; ** P ≤ 0.01; *** P ≤ 0.001). All box plots display median values (middle line) and the lower and upper quartile values form the box, with whiskers extending to one and a half times the interquartile range

The optimized methodology was used to elucidate rosetting characteristics in all four ABO Bgs for a wider panel of parasites. In total, three out of six parasite lines showed significantly increased RR or RS in any of the non-O Bgs compared to BgO (Fig. 3, Additional file 1: Fig. S1). FCR3S1.2 displayed a trend of higher RR in all non-BgO and significantly higher RR in BgAB (79%, IQR 79–82) as compared to BgO (70%, IQR 69–73) (*P* = 0.02) (Fig. 3A). Moreover, rosettes in FCR3S1.2 were significantly larger in BgA and BgAB as compared to BgO (Fig. 3B). The RR in the Ugandan cerebral malaria patient isolate UCM83 was significantly higher in BgA (54%, IQR 54–56, *P* = 0.005), BgB (54%, IQR 53–55, P < 0.001) and BgAB (53%, IQR 52–53, *P* = 0.004) as compared to BgO (49%, IQR 47–50) (Fig. 3C). On the contrary, for PAvarO, UKM74, UKM104 and UKS111, no substantial difference in RR between ABO blood groups was observed (Fig. 3E, Additional file 1: Fig. S1A, C, E). Despite no difference in RR, PAvarO parasites formed significantly larger rosettes in BgA, BgB and BgAB as compared to BgO (Fig. 3F). Besides FCR3S1.2 and PAvarO, no significant difference in RS between blood groups was observed (Additional file 1: Fig. S1B, D, F).

It has been previously shown that parasite Bg preference influences the ease of rosette disruption by heparin [12, 16]. In order to test whether the Bg of the host would affect the sensitivity of rosettes for heparin, the same cultures were treated with 10 μg/mL for 45 min prior to flow cytometry. Indeed, for the A-preferring parasite FCR3S1.2 [12], rosettes were more resistant to heparin when cultivated in BgA and BgAB (Fig. 3G, H). Similar outcomes could be observed for UCM83, with the addition of more stable rosettes also in BgB (Fig. 3I, J), whereas no differences between Bgs were noted for PAvarO (Fig. 3K, L). Similarly, no differences between Bgs to heparin dependent rosette disruption were observed for UKM74, UKM104 and UKS111, except UKM104 having slightly larger rosettes in BgA and surprisingly UKS111 being more susceptible to heparin in BgB (Additional file 1: Fig. S1G–L). To summarize, the influence of ABO blood group on rosetting differ substantially between parasite lines and both A- and B-antigens can augment RR and RS depending on the parasite background.

### Non-O blood groups reduce PfEMP1-DBL1α epitope accessibility

**Fig. 4:**
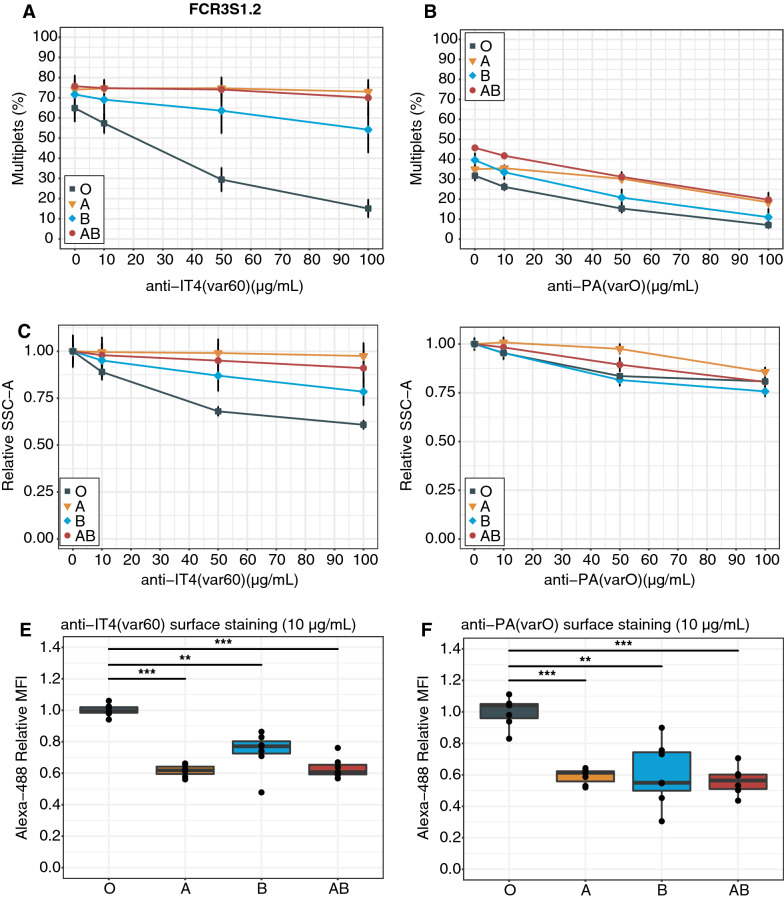
Non-O blood groups reduce PfEMP1-DBL1α epitope accessibility. FCR3S1.2 (left column) and PAvarO (right column) parasites grown in erythrocytes from seven different donors from each blood group were exposed to anti-PfEMP1-DBL1ɑ antibodies for one hour. After treatment, rosetting rate (**A**, **B**) and rosette size (**C**, **D**) was evaluated by flow cytometry in BgO (squares), BgA (triangles), BgB (rhombus) and BgAB (circles) (mean ± SD). **A**, **B** Rosette disruption was measured as the absolute rosetting rate (percentage of multiplets). **C**, **D** Reduction of rosette size is shown as relative decrease of mean multiplets’ SSC-A. Samples were normalized to the mean SSC-A of the untreated samples for each blood group. **E**, **F** The accessibility of PfEMP1 in rosettes by antibodies was measured by immunofluorescence. Samples were normalized to the mean MFI of BgO. Pairwise comparison of BgO and all non-O Bgs performed using unpaired t-test with Holm adjustment of P-values indicated by asterisks (* P ≤ 0.05; ** P ≤ 0.01; *** P ≤ 0.001). All box plots display median values (middle line) and the lower and upper quartile values form the box, with whiskers extending to one and a half times the interquartile range

Previous studies have found that PfEMP1-DBL1α epitopes of FCR3S1.2 and PAvarO are more accessible in BgO than BgA, with rosettes being more sensitive to antibody-induced dispersion in BgO [17]. To expand on those findings to all ABO blood groups, strain-specific polyclonal PfEMP1-DBL1α antibodies at concentrations of 0, 10, 50 and 100 μg/mL were added to cultures grown in all Bgs and rosetting characteristics were measured by flow cytometry. An incremental rosette disruption was observed with higher antibody concentrations, but in a strong Bg dependent manner (Fig. 4A–D). For the parasite FCR3S1.2, antibodies were more potently disrupting rosettes formed with BgO, followed by BgB and BgAB whereas the effect on BgA rosettes was close to non-existent (Fig. 4A, C). PAvarO parasites showed a similar trend upon addition of a strain-specific antibody, still with BgO being most susceptible to rosette disruption but less prominently so compared to FCR3S1.2 (Fig. 4B, D).

The accessibility of PfEMP1 to antibodies in the different Bgs was further evaluated. To minimize the rosette disruptive effects of antibodies the lowest tested concentration was chosen (10 μg/mL). After incubation with PfEMP1-DBL1α antibodies, cells were stained with fluorescent secondary antibodies. MFI of iRBCs were determined by flow cytometry and the samples were normalized to the mean of the BgO samples. A reduction of antibody accessibility was observed in all non-O Bgs for both parasite lines (Fig. 4E, F). For FCR3S1.2, the antibody accessibility to PfEMP1 in BgA, BgB and BgAB was reduced by 38% (95% CI 35–42), 26% (95% CI 21–31) and 37% (95% CI 31–43), respectively as compared to BgO (Fig. 4E). Similarly, in PAvarO a 41% (95% CI 36–45) reduction in antibody accessibility was observed in BgA, 39% (95% CI 21–58) in BgB and 44% (95% CI 34–54) in BgAB (Fig. 4F). In parallel, non-immune goat IgG was used as control and showed no differences between the four blood groups (Additional file 1: Fig. S2). Thus, for parasites being of a Bg dependent rosetting phenotype, non-O Bgs confer protection from rosette disruption and recognition of the mediating parasite adhesins.

### Low BgA-expression levels make rosettes more susceptible to disruptive agents

As erythrocytic BgA antigen expression levels vary significantly between A1 and non-A1 Bgs, the effect of quantitative A-antigen differences on inherent rosetting characteristics as well as heparin- and anti-PfEMP1-DBL1α antibody induced rosette dispersion was investigated. The levels of BgA and BgB antigens were semi-quantified for 126 RBC samples (6 O, 30 A1, 30 non-A1, 30 B and 30 AB) by flow cytometry and normalized to the background MFI of the BgO donors. The mean relative MFI of the A1 RBC samples was 78 (IQR 65–93) as compared to 17 (IQR 11–20) for non-A1 donors, thus being approximately 4.5-fold higher (Additional file 1: Fig. S3A). Further, the mean relative MFI of A-antigen was 35 among BgAB donors (IQR 4–59), but with a clear polarization, indicating the presence of both A-types in the sample population. Substantially smaller relative differences in MFI for B-antigen was observed, with 3- (IQR 2–3) and 2- (IQR 1–2) fold higher abundance compared to BgO for BgB and BgAB, respectively (Additional file 1: Fig. S3B).

**Fig. 5:**
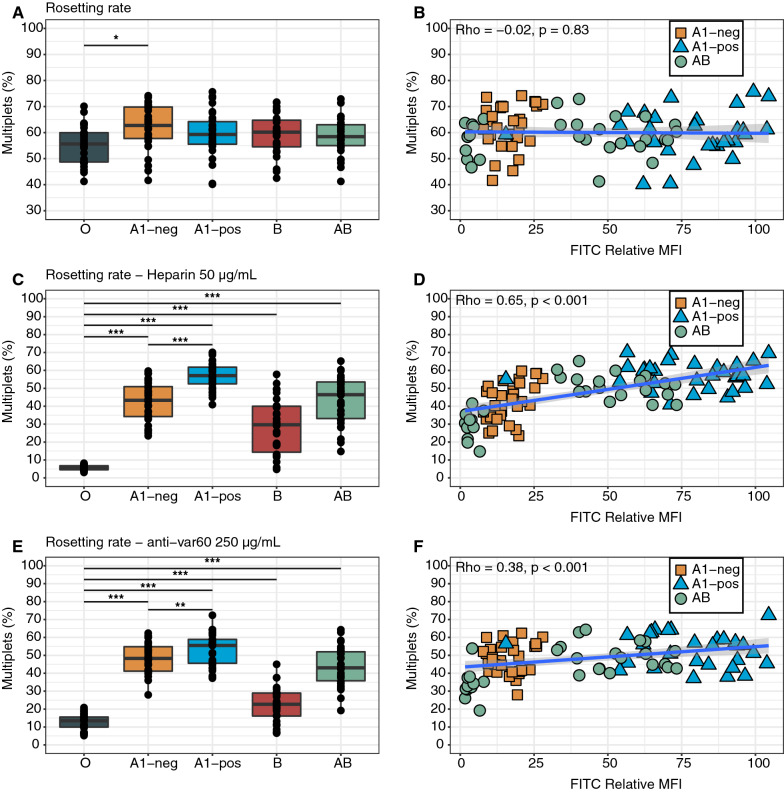
Low erythrocytic BgA-expression levels affects rosetting rate when exposed to rosette-disruptive agents. Rosetting rate of FCR3S1.2 parasites grown in RBCs from 30 donors per blood group (O, A1 negative, A1 positive, B and AB) was measured by flow cytometry in three conditions; **A**, **B** untreated, **C**, **D** heparin 50 µg/mL and **E**, **F** anti-PfEMP1-DBL1ɑ treated (250 µg/mL). Relative A antigen levels were determined by flow cytometry as relative mean fluorescent intensity (MFI) after immunofluorescent staining of RBCs. Correlation between A-antigen levels and rosetting rate was assessed using Spearman’s rank correlation coefficient for each condition (right panels). Pairwise comparison of BgO and all non-O Bgs performed using unpaired t-test with Holm adjustment of P-values (* P ≤ 0.05; ** P ≤ 0.01; *** P ≤ 0.001). All box plots display median values (middle line) and the lower and upper quartile values form the box, with whiskers extending to one and a half times the interquartile range

Using these RBC samples, low A-antigen rosetting could be investigated using the highly rosetting and phenotypically Bg dependent FCR3S1.2. Although rosetting was more prominent in BgA than BgO, no statistically significant differences in RR between A1 negative and positive samples were noted nor any correlation between A-antigen levels and RR (Fig. 5A, B). However, after treatment with heparin (50 µg/mL) for 45 min, there was a clear difference in RR between the BgO and the BgA1 negative, BgA1 positive and BgAB samples (Fig. 5C), with a significant correlation between the BgA antigen quantity and RR (rho = 0.65, p < 0.001) (Fig. 5D).

As rosettes of bulk BgA have been shown to be more resistant to antibody-mediated disruption, a hypothesis arised that, similar to the heparin sensitivity, higher levels of BgA antigen could make the rosettes more resistant [17]. This hypothesis was tested by exposing the samples to 250 µg/mL anti-DBL1α antibodies. Indeed, the A1 positive rosettes were most resistant to dispersion, followed by non-A1 rosettes and AB blood rosettes (Fig. 5E), with a significant positive correlation between the BgA antigen quantity and RR (rho = 0.38, p < 0.001) (Fig. 5F) after antibody treatment.

**Fig. 6:**
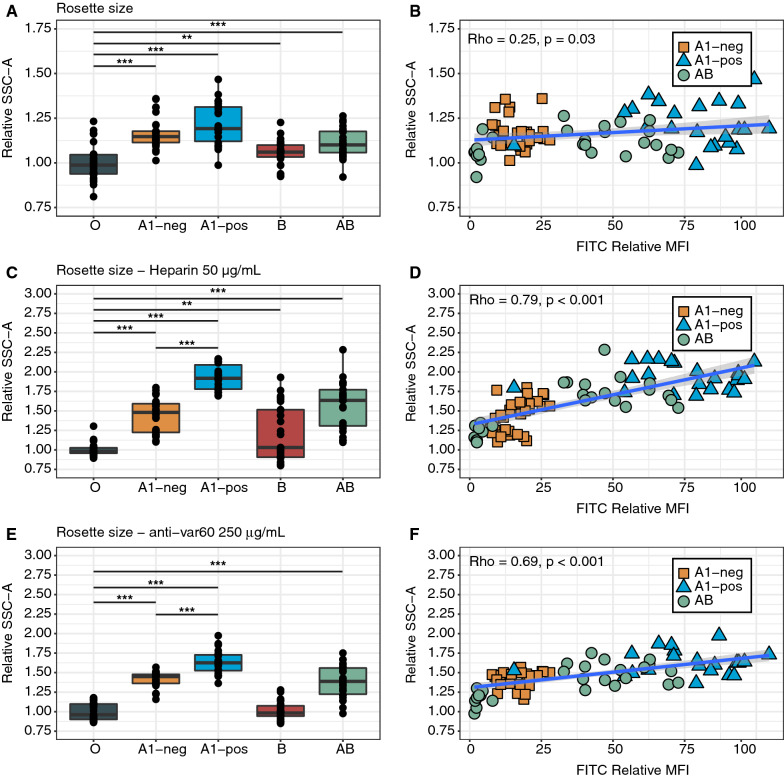
Low erythrocytic BgA-expression levels affects rosette size when exposed to rosette-disruptive agents. Relative rosette size of FCR3S1.2 parasites propagated in RBCs from 30 donors and thereafter exposed to three conditions; **A**, **B** untreated, **C**, **D** heparin 50 µg/mL and **E**, **F** anti-IT4(var60) PfEMP1- DBL1ɑ treated (250 µg/mL). Correlation between A-antigen and rosette size was assessed using Spearman’s rank correlation coefficient for each condition (right panels). Pairwise comparison of BgO and all non-O Bgs performed using unpaired t-test with Holm adjustment of P-values (* P ≤ 0.05; ** P ≤ 0.01; *** P ≤ 0.001). All box plots display median values (middle line) and the lower and upper quartile values form the box, with whiskers extending to one and a half times the interquartile range

Highly similar results were observed for RS, with no clear difference in inherent RS between A1 negative and positive samples (Fig. 6A, B). A significant difference between A1 negative and positive samples and a significant positive correlation between the BgA antigen quantity and RS were observed after heparin- (rho = 0.79, P < 0.001) (Fig. 6C, D) and antibody-treatment (rho = 0.69, P < 0.001) (Fig. 6E, F).

### Serum ABO blood group does not affect parasite rosetting in vitro

**Fig. 7:**
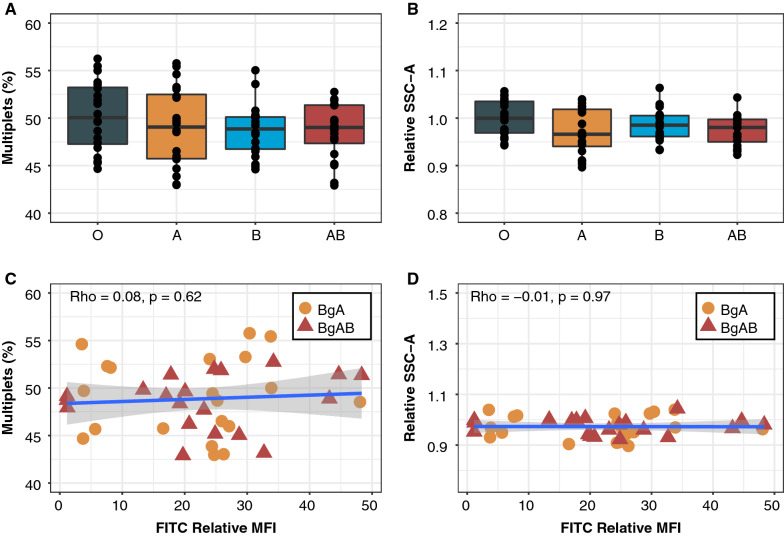
Serum ABO blood group does not affect parasite rosetting in vitro. FCR3S1.2 parasites were grown in O RBCs with sera from 20 donors for each blood group for one life cycle. **A** Rosetting rate and **B** rosette size were measured by flow cytometry. Pairwise comparison of BgO and all non-O Bgs performed using unpaired t-test with Holm-Bonferroni adjustment of P-values. **C**, **D** A antigen levels of RBCs from serum donors were semi-quantified by flow cytometry. Thereafter, the correlation between A-antigen levels and rosetting rate (**C**) and rosette size (**D**) was measured and assessed using Spearman’s rank correlation coefficient. All box plots display median values (middle line) and the lower and upper quartile values form the box, with whiskers extending to one and a half times the interquartile range

As the ABO system possible also affects the serum composition and the role of cognate serum in light of ABO has not been evaluated, an investigation to determine whether serum alone could affect rosetting was undertaken. When comparing rosetting in MCM supplemented with 10% AB^+^-serum as compared to MCM supplemented with 0.5% Albumax, FCR3S1.2 displayed the largest differences in RR and RS (Additional file 1: Fig. S4). Due to the high serum dependency in conjunction with the reliance of host cell resident Bg for the rosetting phenotype, this parasite line was chosen as the point of inquiry. Therefore, FCR3S1.2 parasites were grown in pooled BgO donor RBCs with heat-inactivated serum from 80 different donors (20 of each Bg). The RR and RS were measured at the following erythrocytic cycle by flow cytometry. No difference was observed in RR and RS between the serum of different Bgs (Fig. 7A, B). Further, after measuring relative A antigen levels of RBCs from the serum donors, no correlation was observed between the A antigen levels in BgA and BgAB RBC samples and the RR or RS (Fig. 7C, D). Altogether, these results indicate that the Bg dependent rosetting phenotype is reliant on host cell resident Bg antigens and that serum, in the context of the ABO system, is less influential, at least in the in vitro context.

## Discussion

That ABO Bg influences susceptibility to severe *P. falciparum* malaria and rosetting has been implicated mainly by the previously observed BgO protective effect [17, 33]. Opposite to this, the heterogenous BgA has been suggested as a potential risk factor for SM and although data is limited, there appears to be a more heterogenous distribution of BgA alleles in Africa, which coincides with the highest transmission of *P. falciparum*. The aim of this paper was to gain insights to this by evaluating the role of Bgs in rosetting, a well-known and SM associated virulence feature of the parasite, with an emphasis on the sub-division of BgA [34–36].

Key to this interrogation was the development of a more robust methodology for high-throughput characterization of the rosetting phenotype as the traditional analysis by microscopy is laborious, quantitatively weaker and prone to subjective interpretations. This work led to the possibility of accurately measuring absolute RRs by flow cytometry, which abolished the need for microscopy completely in that respect. For RS however, which is a more heterogenous and less on/off phenotypic read out, the dynamic range of the data prevented absolute measures. Still, the correlation between RS determined by flow cytometry and microscopy was deemed sufficient.

The use of this methodology allowed for a thorough investigation of Bg influence on the rosetting phenotype for a wide panel of parasite strains and clinical isolates using a large number of RBC and serum donor samples. Indeed, an associations between RR and RS and the major blood groups was identified as expected, associations that were further strengthened upon addition of rosette disrupting agents. BgA and BgAB clearly stood out in mediating both elevated RR and RS and protecting against disruptive agents, although with large inter-parasite variations. The latter was expected as the rosetting phenotype is not solely dependent on Bgs but is phenotypically diverse and also entails the use of other receptors on the host RBCs [37]. In fact, the plasticity of the phenotype also goes beyond the use of host cell receptors and also includes a wider repertoire of parasite antigens beyond the PfEMP1 family of proteins. Thus, it is plausible that the parasites used in this study that did not reveal any Bg dependency of their rosetting make use of additional ligands and/or non-Bg RBC receptors. Still, for the Bg dependent parasite lines with known PfEMP1 expression and availability of specific DBL1α antibodies, a robust protection of parasite antigens in BgA rosettes was seen. It has been previously observed that rosettes formed with BgA RBCs have reduced epitope accessibility for antibodies against PfEMP1, but here these findings were extended to all non-O Bgs [17]. PfEMP1 serves as a target of naturally acquired immunity and thus, a reduced epitope accessibility in non-O Bgs might be an important factor underlying the increased risk of contracting severe malaria [17, 38].

Several subtypes of the BgA have been defined, with two major subgroups being A_1_ and A_2_. A_1_ RBCs possess approximately five times more A-antigen than A_2_ RBCs as well as a more of the repetitive A epitope glycolipid motifs [18]. Structural studies of the PfEMP1 RBC binding DBL1α_1_ domain of PAvarO parasites showed a higher binding of A1 RBCs compared to A2 and Ax RBCs [20]. These findings got further support from Goel et al. who demonstrated that FCR3S1.2 forms bigger rosettes with A1 RBCs but no difference in RR [13]. Herein, a dependence of RS on the amount of A-antigen present on RBCs was observed with significantly larger rosettes formed in A1-positive blood. Furthermore, A1-positivity made rosettes more resistant to the disruptive agent heparin and antibodies against PfEMP1. Thus, the data presented here support BgA in general and subtypes of BgA in particular as risk factors for SM. In the view of these findings, one would anticipate a stronger selection of BgO, and within BgA a selection for low antigen A, in malaria endemic regions. Although GWAS studies have confirmed the protective effects of BgO for SM, the relative allele distribution of Bgs between populations in malaria endemic and non-endemic continents are not that striking. This is possibly a result of the phenotypically plastic rosetting phenomenon, where a single family of receptors is likely not enough of a force for stronger evolutionary selection.

Another contributing factor behind the plastic rosetting phenomenon was evaluated, namely the role of human serum. Differences between ABO-Bgs stretch beyond the RBC surface antigens. Variations in the levels of plasma components such as VWF and ICAM-1 have been reported [21, 22, 39]. Further, serum factors have been indicated to form intermolecular bridges between parasite antigens on iRBCs and receptors on surrounding RBCs. However, the current study did not find any significant changes in RR nor RS when exposing iRBCs to sera from different Bg donors. These findings suggest that Bg mediated rosetting fully relies on host cell resident ABO antigens. It is however important to note that this might be influenced by limitations of the in vitro cultivation system in mimicking the more complex in vivo setting, where serum ABO Bgs might still play a more substantial role.

The potential impact of SM on the distribution of different BgA alleles in different continents has been previously suggested but the significance of weak phenotypes has not been properly explored [13, 20]. Past epidemiological studies have been pooling the heterogeneous BgA subgroups into the same category, potentially resulting in an underestimation of risk for severe disease. The future studies ideally should subdivide BgA in order to comprehensively investigate the effect of high and low expressing BgA phenotypes on the risk of contracting SM. This is probably also important beyond the role of Bgs for malaria pathogenesis, as BgA has been associated to adverse outcomes also for other diseases, such as in thromboembolic diseases and more recently for COVID-19 [40, 41].

## Conclusions

Using a high-throughput optimized flow-cytometric methodology to assess rosetting characteristics in an extensive panel of RBC and serum samples, this paper demonstrates that all non-O Bgs shield the parasite surface antigens from strain-specific antibodies towards PfEMP1, as well as a positive correlation between A-antigen levels on RBCs and rosette sturdiness. These results warrant caution against interpreting the results from the heterogeneous BgA phenotype as a single group in epidemiological and experimental studies.

## Supplementary Information

**Additional file 1.** Supplementary material.

## Data Availability

The datasets used during the current study are available from the corresponding author on reasonable request.

## Abbreviations

Bg: Blood group
CR1: Completement receptor 1
GYPC: Glycophorin C
IQR: Interquartile range
MCM: Malaria culture medium
MFI: Mean fluorescence intensity
PAvarO: PaloAltoVarO
PfEMP1: Plasmodium falciparum erythrocyte membrane protein 1
RR: Rosetting rate
RS: Rosette size
SM: Severe malaria
