# Supplementary material for: Red blood cell blood group A antigen level affects the ability of heparin and PfEMP1 antibodies to disrupt Plasmodium falciparum rosettes
Source: Malar J. 2021 Nov 18;20:441. doi: 10.1186/s12936-021-03975-w (PMC8600353; doi:10.1186/s12936-021-03975-w)
Supplement: Supplementary file 1 — Additional file 1. Supplementary material. [file 12936_2021_3975_MOESM1_ESM.docx]

Supplementary Materials for

**Red blood cell blood group A antigen level affects the ability of heparin and PfEMP1 antibodies to disrupt Plasmodium falciparum rosettes**

### **Authors:** Pontus Hedberg^1,2^, Madle Sirel^1^, Kirsten Moll^3^, Mpungu Steven Kiwuwa^4^, Petter Höglund^5^, Ulf Ribacke^1^, Mats Wahlgren^1^

Correspondence to: [mats.wahlgren@ki.se](about:blank)

Table of Contents

Figure S1. Non-immune goat IgG surface staining of FCR3S1.2 and PAvarO 2

Figure S2. Flow cytometric semi-quantification of erythrocytic ABO antigen expression 4

Figure S3. Flow cytometric semi-quantification of erythrocytic ABO antigen expression 5

Figure S4. Flow cytometric semi-quantification of erythrocytic ABO antigen expression 6

#

**Figure S1. Blood group A and B influences parasite rosetting in a strain-dependent manner**. Three Ugandan patient isolates (UKM74, UKM104 and UKS111) were grown in RBCs from seven donors per blood group and rosetting characteristics determined by flow cytometry. Rosetting rate (left panel, percentage of multiplets) and rosette size (right panel, relative SSC-A) was measured without **(A-F)** and with **(G-L)** the addition of 10 µg/mL of heparin. Pairwise comparison of BgO and all non-O Bgs performed using unpaired t-test with Holm adjustment of P-values which are indicated with asterisks (* p ≤ 0.05). Box plots show the median value as the middle line with lower and upper quartile values forming the box, whiskers extend to one and a half times the interquartile range.

#

# Figure S2. Non-immune goat IgG surface staining of FCR3S1.2 and PAvarO. (A) FCR3S1.2 and (B) PAvarO parasites were grown in RBCs from seven blood donors per blood group. They were assayed for the binding of non-immune goat Ig at 10 µg/mL. Samples were normalized to the mean MFI of BgO. No differences between blood groups were observed for neither of the parasites. Pairwise comparison of BgO and all non-O Bgs performed using unpaired t-test with Holm adjustment of p-values. Box plots show the median value as the middle line with lower and upper quartile values forming the box, whiskers extend to one and a half times the interquartile range. (NS=non-significant)

#

# Figure S3. Flow cytometric semi-quantification of erythrocytic ABO

# antigen expression. (A) The blood group A-antigen and (B) blood group B-antigen expression levels of 6 blood group O, 30 blood group A1-negative (neg), 30 blood group A1-positive (pos), 30 blood group B and 30 blood group AB RBC donor samples were semi-quantified by flow cytometry. The mean fluorescence intensity (MFI) was normalized to the BgO mean. Box plots show the median value as the middle line with lower and upper quartile values forming the box, whiskers extend to one and a half times the interquartile range.

#

#

# Figure S4. Flow cytometric semi-quantification of erythrocytic ABO antigen expression. FCR3S1.2, UCM83 and UKM104 parasites were grown in malaria-culture medium supplemented with 10% AB-serum or 0.5% Albumax from ring to trophozoite stage. Rosetting rate (percentage of multiplets) (A) and rosette size (relative SSC-A) (B) of each parasite is shown. (n=4, mean±SD)
